# Compassionate use of recombinant human IL‐7‐hyFc as a salvage treatment for restoring lymphopenia in patients with recurrent glioblastoma

**DOI:** 10.1002/cam4.5467

**Published:** 2022-12-30

**Authors:** Stephen Ahn, Jae‐Sung Park, Heewon Kim, Minkyu Heo, Young Chul Sung, Sin‐Soo Jeun

**Affiliations:** ^1^ Department of Neurosurgery, Seoul St. Mary's Hospital College of Medicine, The Catholic University of Korea Seoul South Korea; ^2^ Genexine, Inc. Seongnam‐si Gyeonggi‐do South Korea

**Keywords:** glioblastoma, IL‐7, immunotherapy, lymphopenia

## Abstract

**Purpose:**

Addressing lymphopenia in cancer patients has been suggested as a novel immunotherapeutic strategy. As interleukin‐7 (IL‐7) is necessary for proliferation of lymphocytes and to increase total lymphocyte count (TLC), IL‐7 therapy has been attempted in various cancers. Here, we describe the clinical results of treatment of recurrent glioblastoma (GBM) with a long‐acting engineered version of recombinant human IL‐7 (rhIL‐7‐hyFc).

**Methods:**

This prospective case series based on compassionate use was approved by the Ministry of Food and Drug Safety in South Korea. Primary outcomes were safety profile and TLC. Secondary outcomes were overall survival (OS) and progression‐free survival (PFS).

**Results:**

Among the 18 patients enrolled, 10 received rhIL‐7‐hyFc with temozolomide, 5 received rhIL‐7‐hyFc with bevacizumab, 1 received rhIL‐7‐hyFc with PCV chemotherapy, and 2 received rhIL‐7‐hyFc alone. Mean TLC of the enrolled patients after the first rhIL‐7‐hyFc treatment increased significantly from 1131 cells/mm^3^ (330–2989) at baseline to 4356 cells/mm^3^ (661–22,661). Higher TLCs were maintained while rhIL‐7‐hyFc was repeatedly administered. Median OS and PFS were 378 days (107–864 days) and 231 days (55–726 days), respectively.

**Conclusion:**

Our study reports that IL‐7 immunotherapy can restore and maintain TLC during treatment with various salvage chemotherapies in recurrent GBM patients without serious toxicity.

## Graphical abstract

The long‐acting engineered version of IL‐7 (rhIL‐7‐hyFc) immunotherapy could restore lymphocyte count and maintain elevated TLC when administered with conventional systemic therapy in recurrent GBM patients without serious adverse events. Combination of rhIL‐7‐hyFc with systemic therapy may provide opportunity of clinical benefit to the patients.

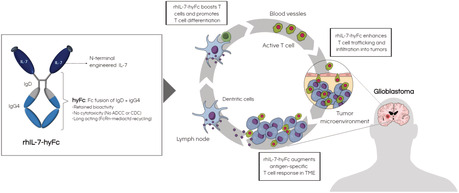

## 1 BACKGROUND

Glioblastoma (GBM) is the most common and devastating brain malignancy in adults.^1^ Median overall survival (OS) is less than 2 years despite aggressive multimodal standard of care including maximal safe resection and concomitant chemoradiation (CCRT) followed by adjuvant chemotherapy with temozolomide.^2^ Tumor recurrence and progression occur in almost all GBM patients, in whom available treatment options include repeat surgical resection; re‐irradiation; and chemotherapies such as temozolomide, bevacizumab, and PCV (procarbazine, lomustine [CCNU], and vincristine). However, the clinical benefit is unsatisfactory.^3^, ^4^

Lymphopenia, which is defined as decreased circulating lymphocytes resulting mainly from radiotherapy, is frequently observed in GBM patients and was recently established as a novel biomarker associated with poor clinical outcomes.^5^, ^6^, ^7^, ^8^, ^9^ Reversing lymphopenia is a novel therapeutic approach in various cancers including GBM.^10^, ^11^ Interleukin‐7 (IL‐7), which is a common gamma‐chain cytokine that plays critical roles in maintaining lymphocyte homeostasis, has been suggested to reverse lymphopenia and improve clinical outcomes of cancer patients.^12^, ^13^ IL‐7 cytokine therapy has been tested in clinical studies of recurrent or refractory cancer, but showed unclear efficacy in terms of T‐cell regeneration and clinical benefit.^14^, ^15^, ^16^, ^17^

In our compassionate use program, our study investigated the clinical benefit of treatment with hybrid‐Fc fused recombinant human IL‐7 (rhIL‐7‐hyFc) in recurrent GBM patients. We evaluated whether rhIL‐7‐hyFc therapy could increase total lymphocyte count (TLC) when used with salvage chemotherapy GBM patients. In addition, we evaluated the safety profile and survival outcomes of enrolled patients.

## 2 METHODS

### 2.1 Ethical considerations

Patients with recurrent GBM received rhIL‐7‐hyFc on a compassionate use basis, as approved and supervised by the Ministry of Food and Drug Safety in South Korea. The study was conducted according to the Declaration of Helsinki and was approved by the institutional review board in Seoul St. Mary's Hospital. It was registered in the National Institutes of Health Clinical Trial Registry (NCT04289155). All patients provided written informed consent prior to compassionate use of rhIL‐7‐hyFc.

### 2.2 Study population

All adult patients who were pathologically confirmed to have recurrent GBM and who were treated at Seoul St. Mary's Hospital were screened. We excluded patients with (a) acute infection, autoimmune, or hematologic disease at the time of recurrence; and (b) expected survival of less than 3 months.

### 2.3 Clinical variables

Baseline characteristics including sex, date of birth, dates of surgeries, pathological findings, prior treatments, and radiological findings were collected and summarized. We also collected data on use of dexamethasone. The concurrent use of dexamethasone was defined as having a prescription of dexamethasone within 2 weeks of rhIL‐7‐hyFc administration. Pathological evaluation was performed by a neuropathologist following the 2016 WHO classification of the central nervous system. Isocitrate dehydrogenase (IDH)1 or IDH2 mutation was assessed by Sanger sequencing. Co‐deletion of 1p19q was identified by fluorescence in situ hybridization. [6]‐Methylguanine‐DNA methyltransferase (MGMT) gene methylation and TERT promoter mutation status were evaluated by polymerase chain reaction (PCR). Loss of ATRX was assessed using immunohistochemistry. Radiographic responses on magnetic resonance imaging (MRI) were determined by two neuroradiologists according to Immunotherapy Response Assessment in Neuro‐Oncology (iRANO) criteria. The duration of recurrence was defined as days from initial surgery to the date of MRI showing recurrence.

### 2.4 Treatment protocols of rhIL‐7‐hyFc therapy

rhIL‐7‐hyFc was prepared and provided by Genexine, Inc. and detailed information for rhIL‐7‐hyFc was previously reported.^18^ In brief, rhIL‐7‐hyFc is a recombinant human IL‐7 fused to the hybridizing IgD/IgG4 immunoglobulin domain to increase the half‐life of IL‐7 in vivo. rhIL‐7‐hyFc was administered intramuscularly into the gluteus muscle and/or deltoid muscle every 4–8 weeks. The dose of rhIL‐7‐hyFC was increased from 60 mcg/kg to 1,440 mcg/kg while monitoring for toxicity.

Patients received salvage systemic therapy with rhIL‐7‐hyFc treatment, if satisfied with criteria. Systemic therapy regimen was determined by the treating clinician. Temozolomide was used for most recurrent cases occurring >6 months upon completion of standard of care. Bevacizumab and chemotherapy with procarbazine, lomustine, and vincristine (PCV) were allowed depending on the clinician's assessment. The dosing schedule of rhIL‐7‐hyFc was determined in consideration of both the lympho‐depletion effect of cytotoxic chemotherapy and the recovery time of lymphocyte counts induced by rhIL‐7‐hyFc. In combination treatment with temozolomide, patients were given rhIL‐7‐hyFc 1 week after initiation of temozolomide. In combination treatment with bevacizumab, the patients were given rhIL‐7‐hyFc within 3 days of bevacizumab. In combination therapy with PCV chemotherapy, rhIL‐7‐hyFc was administered 1 week after completion of PCV.

### 2.5 Primary and secondary endpoints

Primary endpoints were safety and restoration of total lymphocyte count. Treatment‐associated toxicity was evaluated at every visit using the Common Terminology Criteria for Adverse Events (CTCAE) version 5.0. Complete blood count including white blood cells and composition of neutrophils and lymphocytes were calculated at the time of blood sampling on the day of chemotherapy, day of administration of rhIL‐7‐hyFc, and 3 weeks after initial administration of rhIL‐7‐hyFc. Secondary endpoints were overall survival (OS) and progression‐free survival (PFS) after recurrence. OS was also defined as days from the date of MRI showing recurrence before use of rhIL‐7‐hyFc to date of death. Patients alive on February 28, 2021 were censored. The mean duration of follow‐up was 372.6 days (range 98–864). To compare the OS of enrolled patients with historical patients, 123 patients with recurrent GBM diagnosed and treated in this hospital between 2014 and 2017 were enrolled in the historical cohort. The detailed characteristics of the historical cohort are described in Table S1. These patients were followed by routine magnetic resonance imaging (MRI) every 8 or 12 weeks if they did not exhibit neurologic symptoms.

### 2.6 Statistical analysis

Continuous clinical variables such as TLC are expressed as mean value ± standard deviation. Swimmer plot and all figures were constructed using GraphPad Prism software (Version 8.4.3). Kaplan–Meier survival was used to estimate median OS and PFS. Log‐rank test was used to compare OS between the treatment group and historical control. Statistical analysis was estimated using R Statistical Software (Version 4.0.5).

## 3 RESULTS

### 3.1 Characteristics and clinical follow‐up of patients treated with rhIL‐7‐hyFc

**FIGURE 1:**
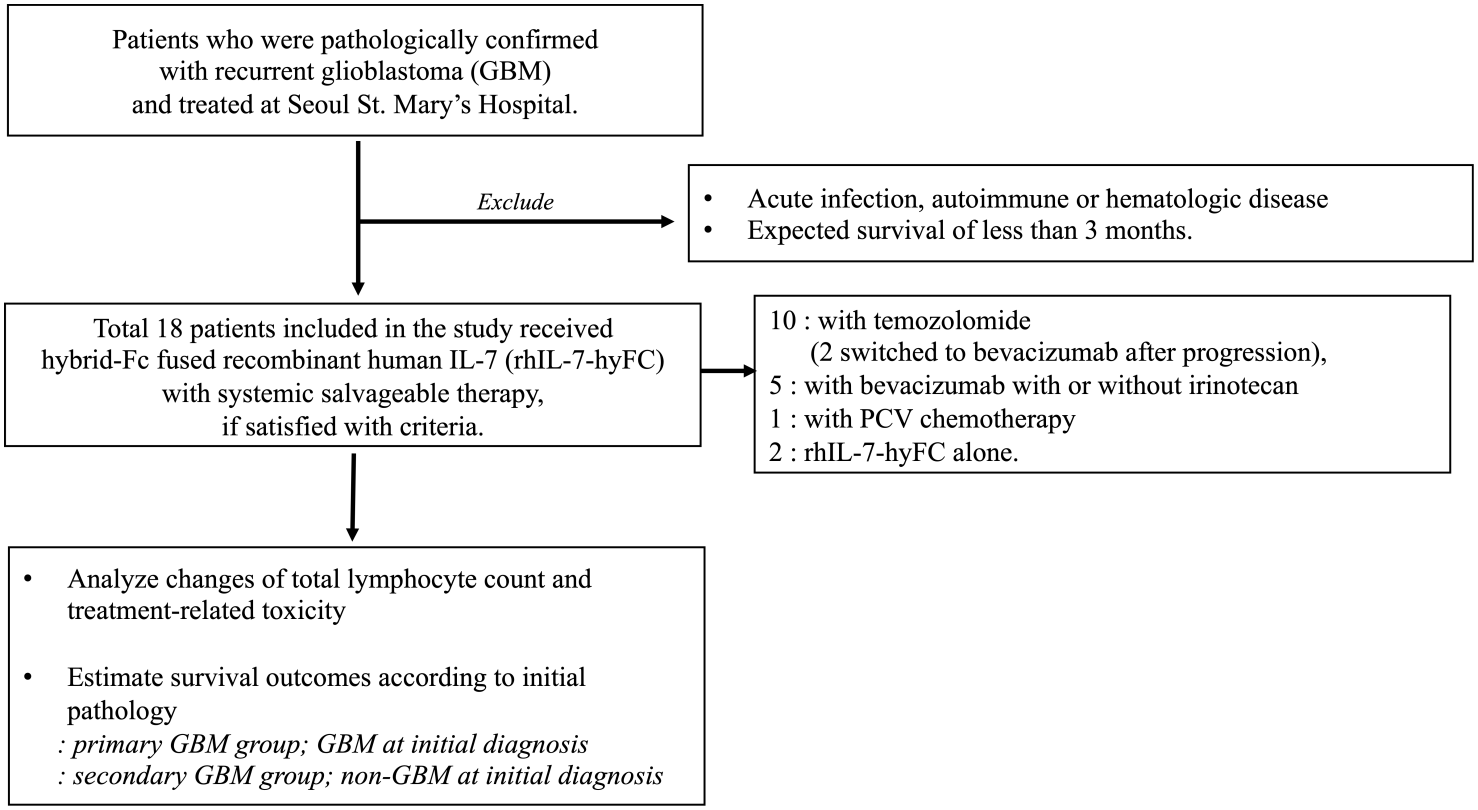
Flow chart of the study design.

Of the 18 patients included in the study, 10 had an initial pathology of GBM (primary GBM group) and 8 had an initial pathology of glioma (secondary GBM group; 3 diffuse astrocytoma and 5 anaplastic astrocytoma). Clinical characteristics of sex, age, pathological findings, the presence of leptomeningeal spread at enrollment, and history of treatment including surgery, chemotherapy, and radiotherapy before enrollment are presented in Table 1. Of 18 patients, 10 started rhIL‐7‐hyFc treatment in combination with temozolomide chemotherapy (2 patients switched to bevacizumab after progression), 5 in combination with bevacizumab with or without irinotecan, 1 with PCV chemotherapy, and 2 received rhIL‐7‐hyFc treatment alone. Of these, 72% of patients (13 of 18) received rhIL‐7‐hyFc at least twice, while 28% of patients (5 of 18) received it only one time. The initial dose was 60 mcg/kg, which was increased to 1440 mcg/kg. Detailed information on rhIL‐7‐hyFc injection with systemic therapy and dexamethasone is presented in Table 2, and clinical follow‐up of the patients is illustrated in Figure 1.

**TABLE 1:** Clinical characteristics of patients with recurrent GBM

						Final molecular markers									
Patient	Age	Sex	Secondary	Initial histology	Final histology	IDH1	IDH2	1p19q	MGMT status	TERT status	ATRX loss	Leptomeningeal spread at enrollment	Time from initial diagnosis to recurrence (days)	Recurrence before rIL‐I7	Treatments before rhIL7‐hyFC
GBM1	37	M	Y	DA	GBM	N	N	N	Y	N	N	Y	1957	1	RT
GBM2	57	F	N	GBM	GBM	N	N	N	Y	UN	N	N	503	1	RT/TMZ
GBM3	74	M	Y	AA	GBM	Y	UN	N	Y	UN	UN	N	1213	1	RT/TMZ
GBM4	66	F	Y	AA	GBM	N	N	N	N	Y	N	N	1254	2	RT/TMZ, Surgery, reTMZ
GBM5	44	F	Y	DA	GBM	N	N	N	N	U	N	N	553	2	RT, PCV
GBM6	54	F	N	GBM	GBM	N	N	N	N	UN	UN	N	241	1	RT/TMZ
GBM7	23	M	N	GBM	GBM	N	N	N	N	UN	Y	Y	921	1	RT/TMZ
GBM8	62	M	N	GBM	GBM	N	N	N	N	Y	N	N	239	1	RT/TMZ
GBM9	69	F	Y	AA	GBM	N	N	N	Y	Y	N	N	132	1	RT, Surgery
GBM10	63	F	Y	AA	GBM	N	N	N	Y	Y	N	N	201	2	RT, TMZ, Surgery
GBM11	51	F	N	GBM	GBM	N	N	N	Y	N	Y	N	418	1	RT/TMZ
GBM12	56	M	N	GBM	GBM	N	N	N	N	Y	N	N	595	1	RT/TMZ
GBM13	74	F	Y	AA	GBM	N	N	N	N	Y	N	N	379	1	RT/TMZ, Surgery
GBM14	54	F	N	GBM	GBM	N	N	N	N	Y	N	N	180	1	RT
GBM15	30	M	N	GBM	GBM	N	N	N	N	UN	N	N	829	2	RT/TMZ, reRT, reTMZ
GBM16	63	F	N	GBM	GBM	N	N	N	N	Y	N	N	251	1	RT/TMZ
GBM17	47	M	N	GBM	GBM	N	N	N	N	N	N	N	649	1	RT/TMZ
GBM18	34	M	Y	DA	GBM	Y	N	N	N	Y	Y	N	2687	2	RT, PCV, Surgery, TMZ

Abbreviations: AA, anaplastic astrocytoma; AO, anaplastic oligodendroglioma; DA, diffuse astrocytoma; GBM, glioblastoma; N, negative; reRT, re‐radiotherapy; reTMZ, re‐temozolomide; RT, radiotherapy; TMZ, temozolomide; UN, unknown.

**TABLE 2:** Details of treatment with rhIL‐7‐hyFc and clinical follow‐up of patients with recurrent GBM

Patients	Number of shots	Dose, mcg/kg (number of hosts)	Treatment in addition to rhIL‐7‐hyFc	Concurrent use of Dexamethasone
GBM1	8	60(5), 240(2), 480(1)^a^	TMZ‐ > Avastin	Cycle 1,7
GBM2	2	120(2)^a^	TMZ	Cycle 1,2
GBM3	4	120 (3), 240 (1)	TMZ	Cycle 2
GBM4	3	120	None	Cycle 1
GBM5	7	240(10), 480(4), 720 (2)	TMZ‐ > Avastin	Cycle 1
GBM6	3	600(2), 720(1)	Avastin + irinotecan	Cycle 1,2
GBM7	3	480(2), 720(1)	TMZ	Cycle 2,3
GBM8	1	720	None	Cycle1
GBM9	12	720	TMZ	Cycle 6,7
GBM10	6	720	TMZ	None
GBM11	5	720	Avastin + Irinotecan	Cycle 6
GBM12	3	720	TMZ	Cycle 4
GBM13	1	960	TMZ	None
GBM14	1	1200	Avastin	Cycle 1
GBM15	1	1200	PCV	None
GBM16	1	1440	Avastin	Cycle 1
GBM17	3	1200	TMZ	None
GBM18	1	1200	Avastin	None

^a^ Injected every 4 week.

### 3.2 TLC changes following treatment with rhIL‐7‐hyFc

**FIGURE 2:**
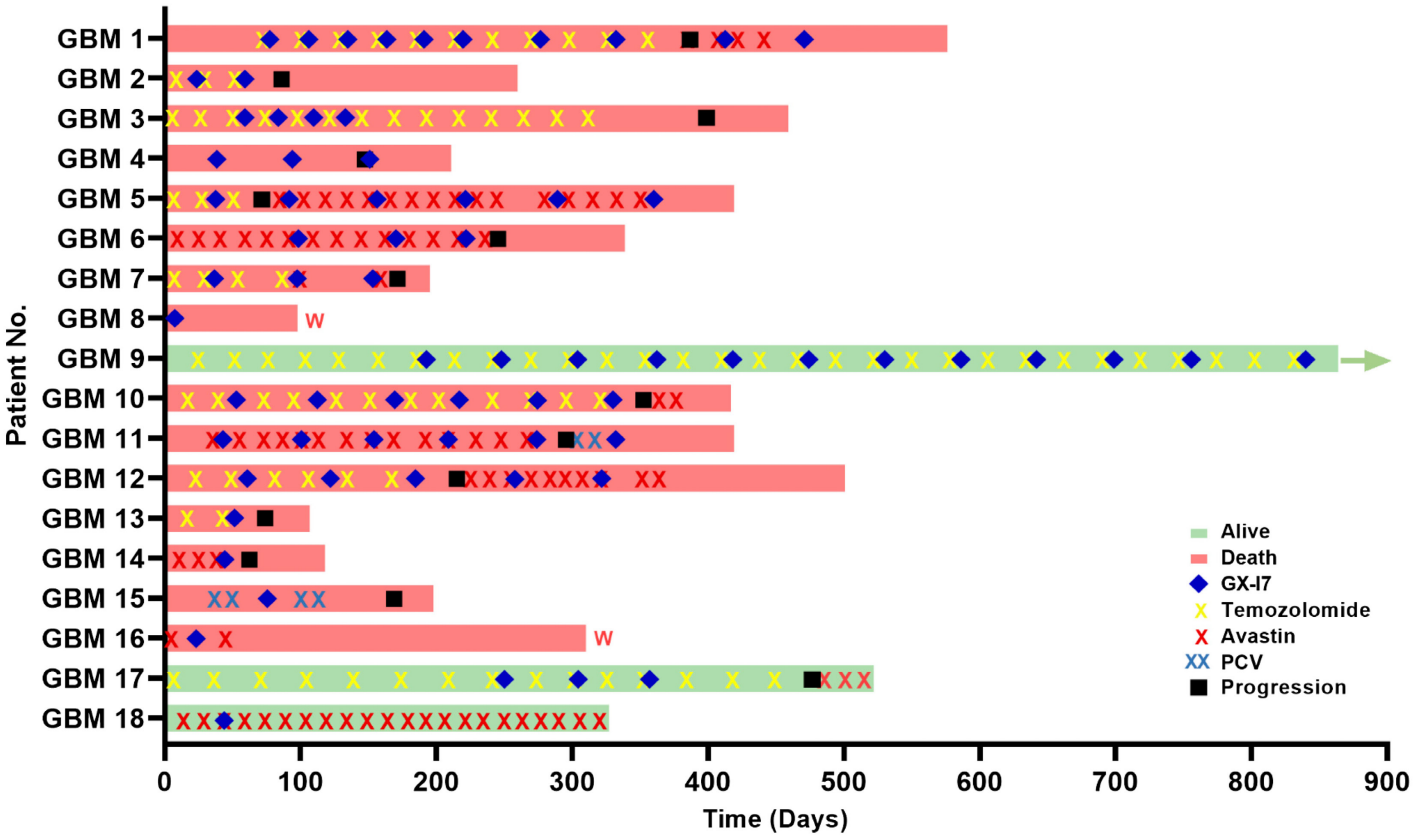
Swimmer plot of the clinical course of patients after enrollment. A total of 18 patients were assessed in this program. Each bar represents an individual patient's treatment history with subsequent treatment and the bar color indicating survival status. These included the following: rhIL‐7‐hyFc with temozolomide, *n* = 10; rhIL‐7‐hyFc with bevacizumab, *n* = 5; rhIL‐7‐hyFc with PCV chemotherapy, *n* = 1; rhIL‐7‐hyFc alone, *n* = 2).

Mean TLC of patients before treatment with rhIL‐7‐hyFc was 1131 (range 330–2989) cells/mm^3^. After initial treatment with rhIL‐7‐hyFc, mean TLC increased to 4356 (range 661–22,661) cells/mm^3^, a mean 3.40 (range 1.38–9.13)‐fold increase. Increased TLC was noted in 16 of 18 patients (94.4%) after the first treatment, and was maintained following repeated treatment; only one patient (GBM 17) did not respond to rhIL‐7‐hyFc. The changes in TLC in all enrolled patients following injection of rhIL‐7‐hyFc are described in Table S2. The dose of rhIL‐7‐hyFc was categorized as low (60–240 mcg/kg), medium (480–720 mcg/kg), and high (960–1440 mcg/kg); TLC significantly increased in a dose‐dependent manner for all patients but one (GBM17), as denoted in Figure 2.

### 3.3 Safety profile and survival outcomes

Most common adverse events related to rhIL‐7‐hyFc were injection site reactions including urticaria, redness, and/or itching sensation near the injection site (64.7%). Of these patients, only one showed grade 3 urticaria and itching sensation. Other serious adverse events (grade 3–4) observed included edema near the injection site (13.2%), fatigue (2.9%), and febrile sensation (2.9%) (Table 3).

**TABLE 3:** Adverse events to treatment with rhIL‐7‐hyFc

Adverse events	Number of adverse events (≥Grade III)	
Urticaria, redness, and itching sensation at near injection site	44 (1)	64.7% (1.47%)
Edema near injection site	9 (0)	13.2%
Fatigue	2 (0)	2.94%
Febrile sensation	2 (0)	2.94%

**FIGURE 3:**
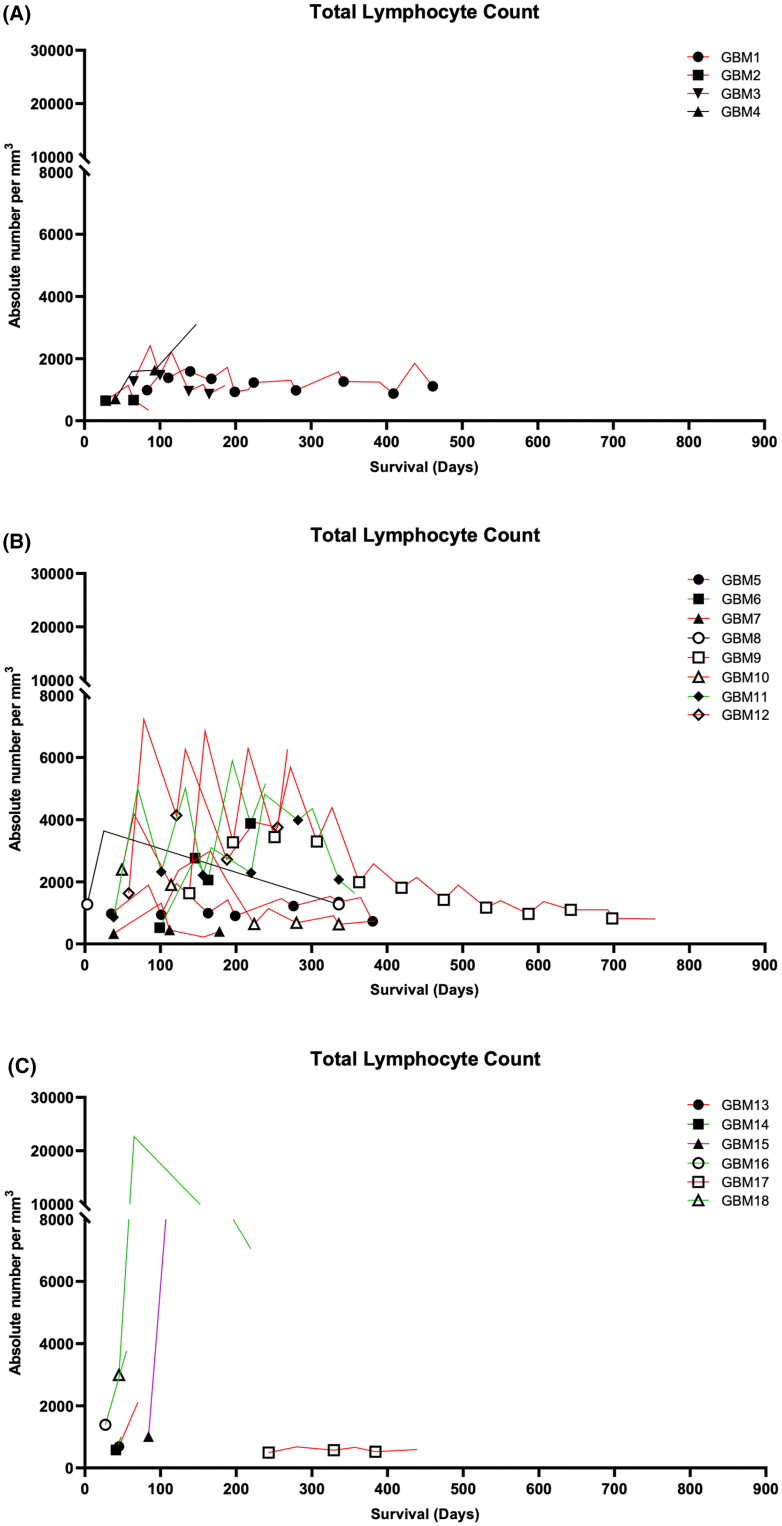
Changes in total lymphocyte count following treatment with rhIL‐7‐hyFc. Patients were treated with 120–240 mcg/kg rhIL‐7‐hyFc (A), 480–720 mcg/kg rhIL‐7‐hyFc (B) and 960–1440 mcg/kg rhIL‐7‐hyFc (C). Symbol: rhIL‐7‐hyFc administration, red line: TMZ treatment in addition to rhIL‐7‐hyFc, green line: avastin or avastin+irinotecan treatment in addition to rhIL‐7‐hyFc, purple line: PCV treatment in addition to rhIL‐7‐hyFc.

We estimated survival outcomes according to initial diagnosis (primary GBM group and secondary GBM group). Median OS of the primary and secondary GBM groups was 285.0 days (range 98–522) and 439.0 days (range 107–864), respectively (Figure 3A). Median PFS of the primary and secondary GBM groups after recurrence was 196.5 days (range 55–301 days) and 372.5 days (65–726 days), respectively (Figure 3B).

**FIGURE 4:**
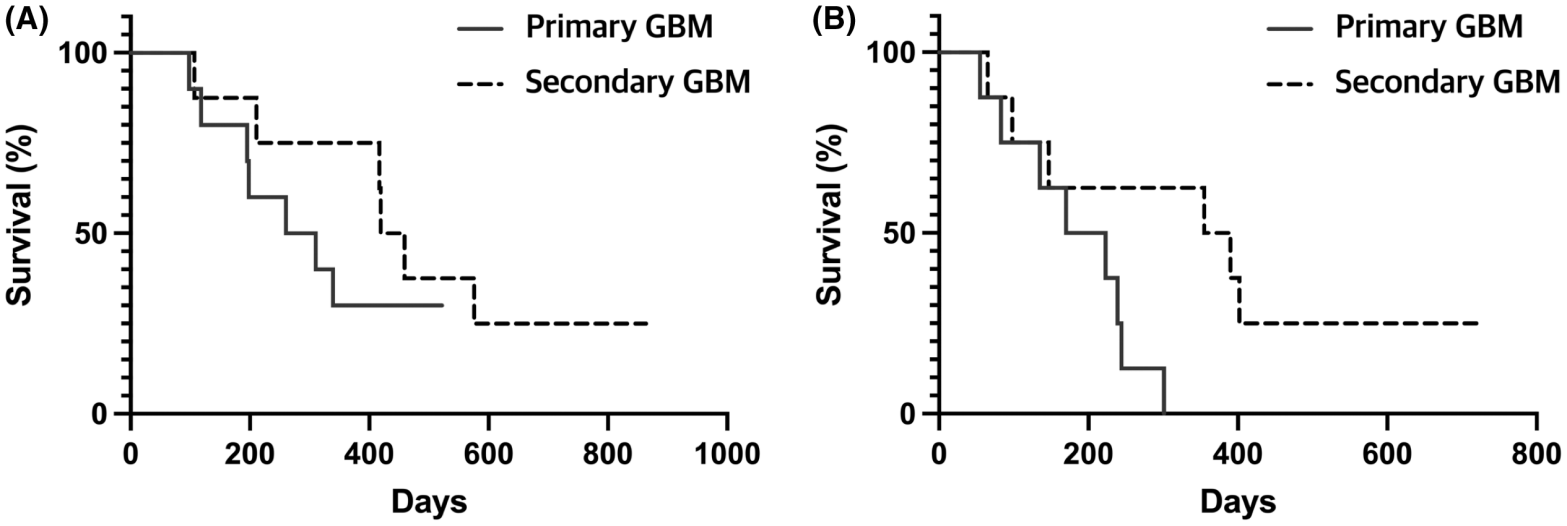
Kaplan–Meier survival curve for overall survival (A) and progression‐free survival (B) of the primary and secondary GBM groups.

Of the 10 patients in the primary GBM group, 7 (70.0%) survived more than 6 months and 3 (30.0%) survived more than 1 year after treatment with rhIL‐7‐hyFc. In addition, four patients (40.0%) had stable disease for more than 6 months after co‐treatment with rhIL‐7‐hyFc injection and systemic therapy. Of the eight patients in the secondary GBM group, seven (87.5%) survived more than 6 months and six (75.0%) survived more than 1 year after treatment with rhIL‐7‐hyFc. In addition, five patients (62.5%) had stable disease for more than 6 months after co‐treatment with rhIL‐7‐hyFc injection and systemic therapy. Among them, two patients (GBM6 & 11) co‐treated with bevacizumab had a partial response, and one patient (GBM9) co‐treated with temozolomide had stable disease for more than 2 years after treatment with rhIL‐7‐hyFc injection (Figure S1). Representative radiographic findings from these patients are presented in Figure 4.

## 4 DISCUSSION

We demonstrated that rhIL‐7‐hyFc can be safely added to conventional salvage systemic therapy such as temozolomide, bevacizumab, and PCV chemotherapy. There are concerns about whether rhIL‐7‐hyFc can restore lymphocyte count when used in combination with cytotoxic chemotherapy, but our study showed that the TLCs of patients co‐treated with rhIL‐7‐hyFc and chemotherapy increased dramatically from 1131 (range 330–2989) cells/mm^3^ at baseline to 4356 (range 661–22,661) cells/mm^3^ after the first treatment, a mean 3.40‐fold increase (range 1.38–9.13). The increase in TLC was dose‐dependent. In addition, continuing rhIL‐7‐hyFc therapy facilitated recovery from lymphopenia induced by concurrent chemotherapy, and this higher TLC was maintained throughout treatment. Mean OS after recurrence of rhIL‐7‐hyFc‐treated patients exceeded 1 year (378 days total, 285 days in the primary GBM group, and 439 days in the secondary GBM group), with two patients showing partial response and one patient showing stable disease for more than 2 years. Because of the small number of patients (*n* = 18) and the potential impact of salvage systemic therapy, it is premature to draw any conclusions; therefore, a larger randomized controlled trial is needed to validate these results. However, our study highlights that rhIL‐7‐hyFc cytokine therapy effectively and safely restored lymphocyte count and ongoing treatment sustained TLC in spite of concurrent systemic therapy with temozolomide, bevacizumab, or PCV chemotherapy in recurrent GBM patients. In addition, concurrent use of dexamethasone, which relieves brain edema and improves neurological condition, did not seem to reduce the efficacy of rhIL‐7‐hyFC cytokine therapy. Detailed information on concurrent use of dexamethasone is provided in Table S2.

Ample evidence suggests that lymphopenia is one of the poorest prognostic factors in various cancer types including GBM.^5^, ^6^, ^7^, ^8^, ^10^ The standard of care for GBM includes aggressive concurrent chemoradiation followed by chemotherapy using temozolomide, which causes severe and prolonged lymphopenia in most GBM patients.^11^, ^19^ In this context, preventing or reversing lymphopenia has been suggested as a novel immunotherapeutic strategy.^5^, ^9^, ^10^ IL‐7, which was first discovered in the 1980s, is a cytokine that elicits T‐cell response to target cancer cells.^13^ It promotes lymphocyte development in the thymus and maintains homeostasis of naive and memory T cells in the periphery.^20^, ^21^ Furthermore, IL‐7 can repair T‐cell injury in cancer patients and overcome an immunosuppressive tumor microenvironment.^11^, ^22^ Several preclinical studies have demonstrated the efficacy of IL‐7 in various cancer types with or without combined therapeutic agents.^23^, ^24^, ^25^

Rosenberg et al. first reported that IL‐7 cytokine therapy administered every 3 days for 8 sessions could increase CD4+ and CD8+ T cells in cancer patients.^15^ In another clinical trial, recombinant human IL‐7 injection every other day for 2 weeks in refractory cancer patients increased CD3+, CD4+, and CD8+ lymphocytes in a dose‐dependent manner.^16^ In another randomized placebo‐controlled phase IIa clinical study, patients with metastatic breast cancer who received three injections of recombinant IL‐7 for 3 weeks had increased CD4 T‐cell counts compared to patients who received placebo.^14^ Lastly, a recent clinical trial using rh‐IL‐7 as adjuvant therapy every 2 weeks combined with dendritic cell vaccination or autologous lymphocyte infusion led to significantly better OS in pediatric sarcoma patients compared to that of the patients in a historical control cohort.^17^ Compared to previous clinical studies of short‐acting IL‐7, our study showed that rhIL‐7‐hyFc could increase TLC drastically even after a single injection. In addition, we demonstrated that rhIL‐7‐hyFc therapy could be combined with conventional systemic therapy such as temozolomide, bevacizumab, or PCV chemotherapy. Even for patients receiving lympho‐depleting cytotoxic chemotherapy, TLC increased after rhIL‐7‐hyFc injection. Therefore, a combination of rhIL‐7‐hyFc with conventional systemic therapies such as temozolomide, bevacizumab, or PCV chemotherapy can provide clinical benefit to patients. To the best of our knowledge, our study is the first to report clinical experience with rhIL‐7‐hyFc therapy in GBM patients. Our preliminary clinical findings will facilitate the development as a novel combination immunotherapy strategy for various cancers including GBM. Considering that lymphopenia is usually a result of concurrent chemoradiation, IL‐7 administered during or after concurrent chemoradiation might be an alternative to prevent or reverse lymphopenia. In addition, increasing lymphocyte counts can augment the anti‐tumor effects of immune checkpoint inhibitors for typical immunotherapy non‐responsive GBM.^10^, ^26^

Our study had several limitations. First, there was selection bias because compassionate use is designed without strict conditions for enrollment. Second, the varied interval between the timing of recurrence and the first injection of rhIL‐7‐hyFc could complicate interpretation of the study. Third, only a few patients who had recurrent primary high‐grade glioma were included, although all patients had pathologically confirmed GBM. Fourth, our study did not include changes in immune subsets including various T‐cell populations such as CD3‐, CD4‐, CD8‐positive T cells, or regulatory T cells. Further prospective studies including immune subsets from peripheral blood and/or tumor tissue are warranted. Fifth, the magnitude of TLC seemed to decline as the cycle repeated in some patients (e.g., GBM 9 and 10). Further studies evaluating antibodies against rhIL‐7‐hyFc are needed. Lastly, we sought to analyze the association between TLC increase and OS, but the results were not significant, possibly because of the small number of patients, varied dose, or heterogeneous clinical groups. Further prospective studies including information on tumor infiltrating lymphocyte counts after rhIL‐7‐hyFc injection are needed to elucidate the exact mechanisms of IL‐7 cytokine therapy in GBM patients.

In conclusion, our study found that long‐acting IL‐7 immunotherapy can restore and maintain TLC when administered with systemic therapy in recurrent GBM patients without serious adverse events. A combination of rhIL‐7‐hyFc with conventional systemic therapy such as temozolomide, bevacizumab, or PCV chemotherapy could provide clinical benefit to patients with cancer. This outcome warrants further large, randomized clinical trials to validate the clinical benefits of rIhL‐7‐hyFc in GBM patients.

## AUTHOR CONTRIBUTIONS

**Stephen Ahn:** Data curation (supporting); writing – original draft (lead). **Jae‐Sung Park:** Supervision (supporting). **Heewon Kim:** Data curation (lead). **Minkyu Heo:** Data curation (supporting). **Young Chul Sung:** Supervision (lead). **Sin‐Soo Jeun:** Conceptualization (equal); supervision (equal).

## CONFLICTS OF INTEREST

Heewon Kim, Minkyu Heo, and Young Chul Sung are employees of Genexine Inc., Korea. None of the other authors have competing interests to declare.

## CONSENT FOR PUBLICATION

Patients provided written informed consent regarding publishing their data and photographs.

## ETHICS APPROVAL AND CONSENT TO PARTICIPATE

This study was conducted according to the Declaration of Helsinki and was approved by the institutional review board at Seoul St. Mary's Hospital. All patients provided written informed consent prior to compassionate use of rhIL‐7‐hyFc.

## Supporting information


Table S1.
Table S2.

Figure S1.

## Data Availability

Raw data that support the findings of this study are avaialbe from the corresponding author, upon reasonable request.
