# Supplementary material for: Compassionate use of recombinant human IL‐7‐hyFc as a salvage treatment for restoring lymphopenia in patients with recurrent glioblastoma
Source: Cancer Med. 2022 Dec 30;12(6):6778–87. doi: 10.1002/cam4.5467 (PMC10067043; doi:10.1002/cam4.5467)
Supplement: Supplementary file 1 — Table S1. Table S2. [file CAM4-12-6778-s001.docx]

**Supplementary Table 1. Changes of total lymphocyte counts following treatments.**

| Patient Code | Cycle | Dose, mcg/kg | WBC (lymphocyte %)  at minimum | TLC  at minimum | WBC (lymphocyte %)  at maximum | TLC  at maximum | Fold  change |
| --- | --- | --- | --- | --- | --- | --- | --- |
| GBM1 | 1* | 60 | 7260 (13.6%) | 987 | 7680 (21.2) | 1,628 | 1.65 |
|  | 2* | 60 | 6030 (22.9) | 1381 | 5720 (29.0) | 1659 | 1.20 |
|  | 3* | 60 | 5710 (27.8) | 1587 | 4670 (29.3) | 1368 | 0.86 |
|  | 4* | 60 | 5090 (26.5) | 1349 | 5010 (34.3) | 1718 | 1.27 |
|  | 5* | 60 | 4200 (22.1) | 928 | 4150 (24.1) | 1000 | 1.08 |
|  | 6 | 240 | 4840 (25.4) | 1229 | 6800 (19.1) | 1299 | 1.00 |
|  | 7 | 480 | 7770 (12.6) | 979 | 9730 (16.2) | 1576 | 1.61 |
|  | 8 | 240 | 6250 (20.2) | 1263 | 5450 (22.9) | 1248 | 0.99 |
|  | 9 | 600 | 3180 (27.5) | 875 | 18900 (9.8) | 1852 | 2.12 |
| GBM2 | 1* | 120 | 4240 (15.3) | 649 | 7920 (14.4) | 1140 | 1.76 |
|  | 2* | 120 | 4880 (13.7) | 669 | 5030 (6.8) | 342 | 0.51 |
| GBM3 | 1 | 120 | 5290 (24.0) | 1270 | 5520 (43.8) | 2418 | 1.90 |
|  | 2 | 120 | 4410 (33.3) | 1469 | 6250 (35.5) | 2219 | 1.51 |
|  | 3 | 120 | 7030 (13.5) | 949 | 5320 (22.0) | 1170 | 1.23 |
|  | 4 | 240 | 4740 (18.1) | 858 | 4520 (25.2) | 1139 | 1.33 |
| GBM4 | 1 | 120 | 3640 (19.2) | 699 | 4640 (35.7) | 1592 | 2.28 |
|  | 2 | 120 | 4450 (36.6) | 1692 | N/A | N/A | N/A |
|  | 3 | 120 | 8320 (37.3) | 3103 | N/A | N/A | N/A |
| GBM5 | 1 | 240 | 8550 (11.4) | 975 | 9730 (19.5) | 1897 | 1.95 |
|  | 2 | 480 | 4880 (19.6) | 941 | 3510 (55.5) | 1948 | 2.07 |
|  | 3 | 480 | 2610 (37.9) | 989 | 1600 (49.4) | 790 | 0.80 |
|  | 4 | 480 | 2240 (40.2) | 900 | 5410 (27.0) | 1461 | 1.62 |
|  | 5 | 480 | 5410 (22.5) | 1217 | 5750 (26.6) | 1530 | 1.26 |
|  | 6 | 720 | 8000 (16.9) | 1352 | 6080 (24.5) | 1490 | 1.10 |
|  | 7 | 720 | 3560 (20.5) | 730 | N/A | N/A | N/A |
| GBM6 | 1 | 600 | 2590 (20.1) | 521 | 4670 (59.1) | 2760 | 5.30 |
|  | 2 | 600 | 8770 (23.5) | 2061 | 9770 (60.3) | 5891 | 2.86 |
|  | 3 | 600 | 8120 (47.8) | 3881 | 11870 (43.5) | 5163 | 1.33 |
| GBM7 | 1 | 480 | 2000 (16.5) | 330 | 2790 (23.7) | 661 | 2.00 |
|  | 2 | 480 | 4980 (26.3) | 1310 | 1130 (39.8) | 450 | 0.34 |
|  | 3 | 720 | 2470 (8.9) | 220 | 1900 (13.8) | 400 | 1.82 |
| GBM8 | 1 | 720 | 4990 (25.5) | 1272 | 11530 (31.5) | 3632 | 2.85 |
| GBM9 | 1 | 720 | 4690 (34.8) | 1632 | 12310 (55.6) | 6844 | 4.19 |
|  | 2 | 720 | 6900 (47.4) | 3271 | 11860 (53.0) | 6286 | 1.92 |
|  | 3 | 720 | 7810 (44.0) | 3436 | 11140 (51.0) | 5681 | 1.65 |
|  | 4 | 720 | 8120 (40.6) | 3297 | 9730 (45.1) | 4388 | 1.33 |
|  | 5 | 720 | 7180 (27.7) | 1989 | 7900 (32.7) | 2583 | 1.30 |
|  | 6 | 720 | 5420 (33.4) | 1810 | 5650 (37.9) | 2141 | 1.18 |
|  | 7 | 720 | 4640 (30.6) | 1420 | 5800 (32.7) | 1897 | 1.34 |
|  | 8 | 720 | 4860 (24.1) | 1171 | 5200 (26.8) | 1394 | 1.19 |
|  | 9 | 720 | 3140 (30.9) | 970 | 6480 (21.1) | 1367 | 1.41 |
|  | 10 | 720 | 4660 (23.6) | 1100 | 3590 (30.6) | 1099 | 1.00 |
|  | 11 | 720 | 3710 (22.1) | 820 | N/A | N/A | N/A |
|  | 12 | 720 | 4010 (20.0) | 802 | N/A | N/A | N/A |
| GBM10 | 1 | 720 | 8490 (28.2) | 2394 | 9340 (44.8) | 4184 | 1.75 |
|  | 2 | 720 | 9170 (20.7) | 1898 | 4900 (48.4) | 2372 | 1.25 |
|  | 3 | 720 | 7160 (41.8) | 2993 | 5320 (38.3) | 2038 | 0.68 |
|  | 4 | 720 | 6260 (10.2) | 639 | 3390 (33.6) | 1139 | 1.78 |
|  | 5 | 720 | 6650 (10.2) | 678 | 4590 (19.8) | 909 | 1.34 |
|  | 6 | 720 | 5430 (11.6) | 630 | 13660 (5.4) | 738 | 1.17 |
| GBM11 | 1 | 720 | 1850 (46.5) | 860 | 7140 (70.3) | 5019 | 5.83 |
|  | 2 | 720 | 3510 (66.1) | 2320 | 7320 (68.4) | 5007 | 2.16 |
|  | 3 | 720 | 3690 (59.9) | 2210 | 5240 (59.2) | 3102 | 1.40 |
|  | 4 | 720 | 4170 (54.9) | 2289 | 8820 (54.6) | 4816 | 2.10 |
|  | 5 | 720 | 7660 (52.0) | 3983 | 8120 (53.6) | 4352 | 1.09 |
|  | 6 | 720 | 4100 (50.5) | 2071 | 3840 (42.4) | 1628 | 0.79 |
| GBM12 | 1 | 720 | 5920 (27.5) | 1628 | 13260 (54.5) | 7227 | 4.44 |
|  | 2 | 720 | 7850 (52.7) | 4137 | 12740 (49.1) | 6255 | 1.51 |
|  | 3 | 720 | 6920 (39.3) | 2720 | 7000 (56.0) | 3920 | 1.44 |
|  | 4 | 720 | 8410 (44.6) | 3751 | 11790 (53.1) | 6260 | 1.67 |
| GBM13 | 1 | 720 | 4230 (16.3) | 689 | 12550 (16.8) | 2108 | 3.06 |
| GBM14 | 1 | 1,200 | 8130 (7.1) | 577 | 7600 (13.2) | 1003 | 1.74 |
| GBM15 | 1 | 1,200 | 8250 (12.2) | 1007 | 18800 (48.9) | 9193 | 9.13 |
| GBM16 | 1 | 1,440 | 6230 (22.3) | 1389 | 7830 (48.1) | 3766 | 2.71 |
| GBM17 | 1 | 1,200 | 2530 (19.4) | 491 | 2830 (24.0) | 679 | 1.38 |
|  | 2 | 1200 | 3380 (16.9) | 571 | 2880 (22.9) | 660 | 1.15 |
|  | 3 | 1200 | 3120 (16.7) | 521 | 2880 (20.5) | 590 | 1.13 |
| GBM18 | 1 | 1200 | 5860 (51.0) | 2989 | 26850 (84.4) | 22661 | 7.58 |

** Injected every 4 weeks*

**Supplementary Table 2. Steroid use during rhIL-7-hyFC treatments.**

| **Patients** | **Cycle** | **Daily dose (mg)** | **Start date***  **(day)** | **Discontiuation date***  **(day)** |
| --- | --- | --- | --- | --- |
| GBM 1 | Cycle 1 | 20 | -14 | -14 |
|  |  | 10 | -13 | -12 |
|  |  | 5 | -11 | -10 |
|  | Cycle 7 | 5 | 0 | 0 |
| GBM 2 | Cycle 2 | 15 | 18 | 18 |
|  |  | 20 | 19 | 20 |
|  |  | 10 | 21 | 24 |
| GBM 3 | Cycle 2 | 20 | 24 | 27 |
|  |  | 10 | 28 | 30 |
| GBM 4 | Cycle1 | 5 | -11 | -8 |
|  |  | 10 | -14 | -12 |
| GBM 5 | Cycle 1 | 5 | -5 | -5 |
|  |  | 20 | -4 | -2 |
|  |  | 10 | -1 | 0 |
|  |  | 5 | 1 | 1 |
|  |  | 4.5 | 3 | 3 |
|  |  | 1.5 | 21 | 27 |
|  | Cycle 2 | 30 | -17 | -17 |
|  |  | 15 | -16 | -16 |
|  |  | 10 | -15 | -15 |
|  |  | 25 | -14 | -14 |
|  |  | 15 | -13 | -13 |
|  |  | 20 | -12 | -12 |
|  |  | 10 | -11 | -10 |
|  |  | 5 | -9 | -6 |
|  |  | 15 | -3 | -1 |
| GBM 5 | Cycle 6 | 30 | -10 | -10 |
|  |  | 15 | -9 | -9 |
|  |  | 10 | -8 | -8 |
|  |  | 5 | -6 | -6 |
|  |  | 3 | 22 | 28 |
|  |  | 20 | 29 | 31 |
|  |  | 10 | 32 | 34 |
|  |  | 5 | 35 | 37 |
| GBM 6 | Cycle 1 | 10 | 45 | 58 |
|  | Cycle 2 | 15 | 45 | 45 |
|  |  | 20 | 46 | 46 |
|  |  | 10 | 47 | 48 |
|  |  | 5 | 49 | 49 |
| GBM 7 | Cycle 2 | 15 | -5 | -5 |
|  |  | 20 | -4 | -4 |
|  |  | 10 | -3 | -3 |
|  | Cycle 3 | 15 | -3 | -3 |
|  |  | 20 | -2 | -2 |
|  |  | 10 | -1 | 0 |
|  |  | 5 | 1 | 1 |
|  |  | 20 | 3 | 4 |
|  |  | 15 | 5 | 5 |
|  |  | 10 | 6 | 7 |
|  |  | 10 | 7 | 7 |
|  |  | 5 | 8 | 8 |
|  |  | 20 | 24 | 25 |
|  |  | 5 | 26 | 26 |
| GBM 8 | Cycle 1 | 1.5 | 0 | 0 |
|  |  | 5 | 5 | 6 |
|  |  | 0.5 | -14 | -1 |
| GBM 9 | Cycle 6 | 10 | 30 | 30 |
|  | Cycle 7 | 15 | -1 | -1 |
|  |  | 20 | -1 | 13 |
|  |  | 25 | 13 | 13 |
| GBM 11 | Cycle 6 | 5 | 21 | 35 |
|  |  | 10 | 36 | 36 |
|  |  | 5 | 37 | 37 |
|  |  | 10 | 38 | 39 |
|  |  | 25 | 40 | 41 |
|  |  | 15 | 42 | 43 |
|  |  | 20 | 44 | 44 |
|  |  | 25 | 44 | 44 |
|  |  | 10 | 44 | 44 |
|  |  | 20 | 45 | 45 |
|  |  | 4 | 46 | 47 |
|  |  | 15 | 48 | 49 |
| GBM 12 | Cycle 4 | 20 | 28 | 30 |
| GBM 14 | Cycle 1 | 10 | 10 | 11 |
|  |  | 5 | 12 | 13 |
| GBM 16 | Cycle 1 | 15 | -9 | -9 |
|  |  | 20 | -8 | -8 |
|  |  | 10 | -7 | -6 |

** The set point is the treatment date of rhIL-7-hyFC*
